# Investigation of imaging features in contrast-enhanced magnetic resonance imaging of benign and malignant breast lesions

**DOI:** 10.1007/s11604-024-01551-1

**Published:** 2024-03-20

**Authors:** Kazunori Kubota, Tomoyuki Fujioka, Ukihide Tateishi, Mio Mori, Yuka Yashima, Emi Yamaga, Leona Katsuta, Ken Yamaguchi, Mitsuhiro Tozaki, Michiro Sasaki, Takayoshi Uematsu, Shuichi Monzawa, Ichiro Isomoto, Mizuka Suzuki, Hiroko Satake, Hiroshi Nakahara, Mariko Goto, Mari Kikuchi

**Affiliations:** 1 Department of Radiology, Dokkyo Medical University Saitama Medical Center, 2-1-50 Minamiko-Shigaya, Koshigaya, Saitama 343-8555 Japan; 2 Department of Diagnostic Radiology, Tokyo Medical and Dental University, 1-5-45 Yushima, Bunkyo-Ku, Tokyo, 113-8519 Japan; 3 Department of Radiology, Faculty of Medicine, Saga University, 5-1-1, Nabeshima, Saga City, Saga 849-8501 Japan; 4 Department of Radiology, Sagara Hospital, 3-31 Matsubara-Cho, Kagoshima City, Kagoshima 892-0833 Japan; 5 Division of Breast Imaging and Breast Interventional Radiology, Shizuoka Cancer Center Hospital, Nagaizumi, Shizuoka 411-8777 Japan; 6 Department of Diagnostic Radiology, Shinko Hospital, 1-4-47, Wakinohama-Cho, Chuo-Ku, Kobe City, Hyogo 651-0072 Japan; 7 Department of Radiology, St. Francis Hospital, 9-20, Kominemachi, Nagasaki City, Nagasaki 852-8125 Japan; 8 Department of Diagnostic Radiology, Tokyo Metropolitan Cancer and Infectious Disease Center, Komagome Hospital, 3-18-22 Honkomagome, Bunkyo-Ku, Tokyo 113-8677 Japan; 9 Department of Radiology, Nagoya University Graduate School of Medicine, 65 Tsurumai-Cho, Showa-Ku, Nagoya, Aichi 466-8550 Japan; 10 Department of Radiology, Sagara Hospital Miyazaki, 2-112-1 Maruyama, Miyazaki City, Miyazaki 880-0052 Japan; 11 Department of Radiology, Kyoto Prefectural University of Medicine, 465 Kajii-Cho, Kamigyo-Ku, Kyoto City, 602-8566 Japan; 12 Department of Imaging Center, Cancer Institute Hospital, Japanese Foundation for Cancer Research, 3-8-31 Ariake, Koto-Ku, Tokyo 135-8550 Japan

**Keywords:** Breast magnetic resonance imaging, Gadobutrol, Contrast-enhanced imaging, BIRADS, Benign lesions

## Abstract

**Purpose:**

This study aimed to enhance the diagnostic accuracy of contrast-enhanced breast magnetic resonance imaging (MRI) using gadobutrol for differentiating benign breast lesions from malignant ones. Moreover, this study sought to address the limitations of current imaging techniques and criteria based on the Breast Imaging Reporting and Data System (BI-RADS).

**Materials and Methods:**

In a multicenter retrospective study conducted in Japan, 200 women were included, comprising 100 with benign lesions and 100 with malignant lesions, all classified under BI-RADS categories 3 and 4. The MRI protocol included 3D fast gradient echo T1- weighted images with fat suppression, with gadobutrol as the contrast agent. The analysis involved evaluating patient and lesion characteristics, including age, size, location, fibroglandular tissue, background parenchymal enhancement (BPE), signal intensity, and the findings of mass and non-mass enhancement. In this study, univariate and multivariate logistic regression analyses were performed, along with decision tree analysis, to identify significant predictors for the classification of lesions.

**Results:**

Differences in lesion characteristics were identified, which may influence malignancy risk. The multivariate logistic regression model revealed age, lesion location, shape, and signal intensity as significant predictors of malignancy. Decision tree analysis identified additional diagnostic factors, including lesion margin and BPE level. The decision tree models demonstrated high diagnostic accuracy, with the logistic regression model showing an area under the curve of 0.925 for masses and 0.829 for non-mass enhancements.

**Conclusion:**

This study underscores the importance of integrating patient age, lesion location, and BPE level into the BI-RADS criteria to improve the differentiation between benign and malignant breast lesions. This approach could minimize unnecessary biopsies and enhance clinical decision-making in breast cancer diagnostics, highlighting the effectiveness of gadobutrol in breast MRI evaluations.

## Introduction

Contrast-enhanced breast magnetic resonance imaging (MRI) is widely used in detecting and diagnosing breast cancer, screening high-risk groups, staging, preoperative planning, and assessing treatment effectiveness [1–5]. In the field of imaging diagnosis of breast lesions, contrast-enhanced MRI has demonstrated higher diagnostic capabilities than mammography and ultrasonography. Although breast MRI exhibits high sensitivity, its specificity is insufficient, leading to the issue of false positives [6, 7].

The imaging diagnosis of contrast-enhanced breast MRI follows the Breast Imaging Reporting and Data System (BI-RADS) guidelines, incorporating known terms that suggest benign or malignant findings. Although features of malignant lesions have been extensively evaluated, lexicons characterizing benign lesions are limited to circumscribed round or oval shapes, slow-persistent enhancing patterns, and dark internal septations, among others, often resulting in overlap with malignant findings [8].

Although some reports have suggested patient age, lesion size, location, and T2-weighted signal intensity as indicators for benign or malignant diagnosis, they are not addressed in the BI-RADS assessment criteria [8–11]. Moreover, the exploration of comparing or combining these criteria with BI-RADS to develop a comprehensive approach for distinguishing benign and malignant lesions has been limited.

BI-RADS recommends biopsy for category 4 lesions with a 2% or higher probability of malignancy [8]. However, the importance lies in establishing robust criteria for discerning benign cases from malignant cases because performing biopsies indiscriminately is not ideal. By thoroughly considering the possibility of benign outcomes, providing a solid foundation for evaluation can reduce unnecessary biopsies.

Gadobutrol, a macrocyclic gadolinium-based contrast agent (GBCA) with double the gadolinium concentration of other agents [12], offers superior image quality in breast cancer imaging [13–15]. However, Further research is needed on its impact on breast MRI accuracy.

In this study, we compiled contrast-enhanced MR images of benign and malignant breast lesions using gadobutrol from multiple institutions and aimed to elucidate the imaging features for discriminating between malignant and benign lesions by comparing and analyzing various imaging findings, including the BI-RADS 2013 assessment criteria, along with the clinical characteristics of patients.

## Materials and methods

### Patient selection and clinicopathological factor evaluation

We conducted a retrospective multicenter study involving 11 hospitals in Japan. This study was approved by the Ethical Review Board on Clinical Studies of each participating institution. Many subjects in this study had completed their medical treatment and were difficult to contact; therefore, they opted out by public announcement.

The inclusion criteria were as follows: (1) women with BI-RADS category 3 or 4 lesions on MRI; (2) patients aged 20–69 years; and (3) patients who have been informed of having a malignant lesion, if present. The exclusion criteria were as follows: (1) patients with a history of anaphylactoid or anaphylactic reaction to any contrast media; (2) those with impaired renal function (estimated glomerular filtration rate (eGFR) < 30 mL/min/1.73 m^2^); (3)pregnant or breastfeeding women; (4) patients with a history of treatment for breast cancer; (5) patients undergoing drug therapy for breast cancer; and (6) patients judged for any reason as being ineligible for participation in this study by the investigator.

This study aimed to elucidate the characteristics of benign lesions by collecting several samples, and to achieve this, we included benign and malignant cases in a 1:1 ratio. The number of target subjects was 200 (100 benign and 100 malignant lesions). Among patients with suspected malignant breast lesions by mammography or ultrasonography and MRI with gadobutrol between July 1, 2015 and January 31, 2018 at the participating centers in the study, benign cases that did not violate the exclusion criteria were selected retrospectively from January 31, 2018. The benign cases for the study were selected sequentially from those that underwent MRI examinations with gadobutrol. Additionally, for the purpose of having a control, one malignant case was chosen, specifically the one with the imaging date closest to that of the benign case.

Benign lesions were defined as those with a benign histological diagnosis after MRI examination or without a histological diagnosis of cancer within 1 year. Malignant lesions were defined as all cases with a histological diagnosis of breast cancer within 1 year after MRI.

The following data were examined from the medical records: (1) date of birth, (2) height, (3) weight, (4) history of breast treatment (e.g., surgery, radiation, and chemotherapy), (5) menstrual history, (6) menstrual cycle, (7) date of the onset of most recent menstrual period on MRI, (8) pregnancy history, (9) childbirth history, (10) history of female hormone use including low-dose oral contraceptives at the time of MRI, and (11) histopathology results.

### MRI protocols

**Fig. 1:**
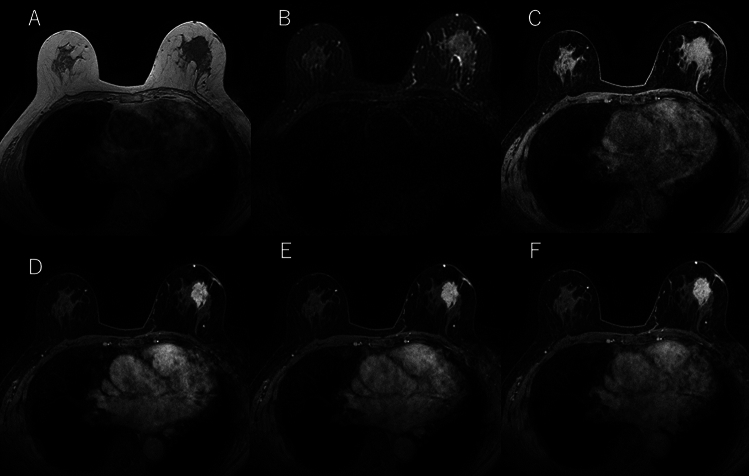
Representative MR images. A represents T1-weighted image, B represents fat-suppressed T2-weighted image, C represents fat-suppressed T1-weighted image, D represents early post-contrast phase, E represents late post-contrast phase, and F represents delayed post-contrast phase

MRI examinations were performed using a 1.5 or 3 T system. 3D fast gradient echo (GRE) T1-weighted images with fat suppression of the entire breasts of either side were acquired with a dedicated breast coil. No restrictions were set regarding the acquisition plane, repetition time, echo time (TE), or flip angle. The prescribed slice thickness was 1–3 mm. Gadobutrol (Gadovist 1.0®, Bayer AG, Germany) was administered at a dose of 0.1 mmol/kg body weight of gadolinium. Dynamic MR images were acquired before and at least two phases, an early phase (1–2 min) and a delayed phase (5–7 min), after bolus injection of the contrast medium, followed by a saline flush using an automatic injector. T1- and fat-suppressed T2-weighted images were also obtained consistently. Figure 1 shows the representative MR images of this study.

### Image analysis

In this study, two radiologists (the first, one of three radiologists with 7–12 years of experience, and the second, a radiologist with 22 years of experience) evaluated breast MR images by consensus without knowledge of the patients’ clinical progress. They assessed lesion characteristics, including lesion location (i.e., side, quadrant, and depth), lesion size, fibroglandular tissue (FGT), background parenchymal enhancement (BPE) (i.e., level and symmetry), signal intensity in T1, and signal intensity in T2. Moreover, for lesions categorized as masses, shape, margin, internal enhancement, and kinetic pattern were evaluated. For lesions classified as non-mass enhancement (NME), they assessed their distribution and internal enhancement patterns. The evaluation of the images was based on the Breast Imaging Reporting and Data System, 5th edition [8]. The medical viewing system EV Insite R (PSP Co., Tokyo, Japan) was used, which offers reading tools, such as window width–window level adaptation, panning, and zooming.

### Statistical analysis

We performed univariate logistic regression analysis, with the case (benign) or control (malignant) group as the dependent variable and clinical and imaging characteristics as the independent variables. Univariate logistic regression analysis was performed by binarizing multiple independent variables wherever possible. Subsequently, we conducted a multivariate logistic regression analysis, with the case (benign) or control (malignant) group as the dependent variable and included age as an adjustment factor. All imaging characteristics that showed a p-value < 0.2 and a sufficient sample size in the univariate logistic regression analysis were entered as independent variables. We then conducted a multivariate decision tree analysis. Furthermore, we devised a predictive model using multivariate logistic regression analysis, and the area under the curve (AUC) was calculated using receiver operating characteristic (ROC) curve analysis. All statistical analyses were performed using Statistical Package for the Social Sciences (version 26; IBM Corp., Armonk, NY, USA), and *p*-values < 0.05 were used to denote statistical significance.

## Results

In our study, all 100 malignant cases underwent histological diagnosis. Of the 100 malignant cases, 66 were diagnosed with invasive ductal carcinoma of no special type, 4 were diagnosed with mucinous carcinoma, 3 were diagnosed with other special types of invasive ductal carcinoma, 3 were diagnosed with invasive lobular carcinoma, and 24 were diagnosed with ductal carcinoma in situ. In the benign group, 91 cases were confirmed as benign using biopsy: 30 fibroadenomas, 24 intraductal papillomas, 14 mastopathies, 8 lobular tumors, 6 sclerosing adenomas, and 9 cases of other histologic types. Among the malignant group, 51 were diagnosed through surgery and 49 through image-guided biopsy, while among the benign group, 29 were diagnosed through surgery, 62 through image-guided biopsy, and 9 through follow-up observation.

Table 1 shows patient characteristics, and Table 2 summarizes the imaging findings. Table 3 presents the results of the univariate analysis, revealing significant differences in independent variables, such as age, lesion location (i.e., quadrant and depth), mass (i.e., shape, margin, internal enhancement, kinetic-initial phase, and kinetic-delayed phase), NME distribution, FGT, BPE level, and T2 signal intensity.

**Table 1:** Patient characteristics

		Benign	Malignant
Age (year)	Mean ± SD	45.8 ± 11.7	54.2 ± 10.3
Height (cm)	Mean ± SD	157.8 ± 4.9	157.7 ± 6.1
Weight (kg)	Mean ± SD	53.2 ± 7.8	56.8 ± 8.3
BMI	Mean ± SD	21.4 ± 3.0	22.9 ± 3.4
Presence of menstruation	Present/absent/unknown	41/27/32	26/55/19
Pregnancy history	Yes/no/unknown	37/48/15	27/57/16
Childbirth history	Yes/no/unknown	41/46/13	29/72/9
History of female hormone use	Yes/no/unknown	7/91/2	8/90/2

BMI, body mass index; SD, standard deviation

**Table 2:** Summary of imaging findings

		Benign	Malignant
Lesion location—Side	Left/right	52/48	54/46
Lesio location—Quadrant	Central	17	2
	LOQ/UOQ/UIQ/LIQ	17/36/20/10	13/45/32/8
Lesio location—Depth	Ant/mid/post	35/7/8	17/34/49
Lesion size (mm)	Mean ± SD	20 ± 19	18 ± 11
FGT	Fatty/scattered/heterogeneous/dense	0/20/49/31	2/32/51/15
BPE—Level	Minimal/mild/moderate/marked	46/32/17/5	60/33/6/1
BPE—Symmetry	Symmetric/asymmetric	93/7	98/2
Signal intensity T1	Hypo/iso/hyper	11/83/6	8/86/2
Signal intensity T2	Hypo/iso/hyper	3/47/50	2/71/27
Lesion—Type	Mass/NME	77/23	74/26
Mass—Shape	Oval/round/irregular	37/14/26	13/8/53
Mass—Margin	Circumscribed/irregular/spiculated	68/9/0	17/40/17
Mass—Internal enhancement	Homogeneous	37	10
	Heterogeneous	34	54
	Rim enhancement	3	10
	Dark internal septation	3	0
Mass—Kinetic initial phase	Fast/medium/slow	19/17/41	4/17/53
Mass—Kinetic delayed phase	Persistent/plateau/washout	48/20/19	23/37/14
NME—Distribution	Focal/linear/segmental/regional	13/1/7/2	6/1/19
NME—Internal enhancement patterns	Homogeneous/heterogeneous/clumped/clustered ring	7/11/4/1	12 /13 / 5 / 3

FGT, fibroglandular tissue; BPE, background parenchymal enhancement; NME, non-mass enhancement; LOQ, left outer quadrant; UOQ, upper outer quadrant; UIQ, upper inner quadrant; LIQ, left inner quadrant; SD, standard deviation

**Table 3:** Univariate analysis

		Regression coefficient	Odds ratio	95% confidence interval		*P*-value
				Lower limit	Lower limit	
Age (years)	Continuous variable	0.068	1.070	1.041	1.101	< 0.001
Lesion size (mm)	Continuous variable	− 0.012	0.988	0.969	1.008	0.244
Lesion location—Side	Left versus right	0.080	1.084	0.622	1.888	0.777
Lesion location—Quadrant	LOQ, UOQ, UIQ, and LIQ versus central	2.306	10.036	2.253	44.712	0.002
Lesion location—Depth	Post versus ant and mid	0.904	2.471	1.374	4.442	0.003
FGT	Fatty, scattered, and heterogeneous versus dense	0.934	2.546	1.273	5.093	0.008
BPE—Level	Minimal and mild versus moderate and marked	1.321	3.747	1.520	9.237	0.004
BPE—Symmetry	Symmetric versus asymmetric	1.305	3.688	0.747	18.211	0.109
Signal intensity T1*	Iso, hypo, hyper	–	–	–	–	0.770
Signal intensity T2	Iso versus hypo, hyper	1.016	2.761	1.540	4.950	0.001
Mass—Shape	Irregular versus oval and round	1.599	4.951	2.479	9.887	< 0.001
Mass—Margin	Irregular and spiculated versus circumscribed	3.232	25.333	10.494	61.155	< 0.001
Mass—Internal enhancement	Heterogeneous and rim enhancement versus homogeneous and dark internal septations	1.934	6.919	3.101	15.437	< 0.001
Mass—Kinetic initial phase	Medium and fast versus slow	1.746	5.733	1.846	17.800	0.003
Mass—Kinetic delayed phase	Plateau and washout versus persistent	1.300	3.670	1.870	7.204	< 0.001
NME—Distribution	Linear and segmental versus focal and regional	1.833	6.250	1.786	21.868	0.004
NME—Internal enhancement patterns	Heterogeneous, clumped, and clustered ring versus homogeneous	0.608	1.837	0.491	6.873	0.366

FGT, fibroglandular tissue; BPE, background parenchymal enhancement; NME, non-mass enhancement; LOQ, left outer quadrant; UOQ, upper outer quadrant; UIQ, upper inner quadrant; LIQ, left inner quadrant

^*^T1 signal intensity could not be binarized

^*^Statistical significance was set at *P* < 0.05

**Fig. 2:**
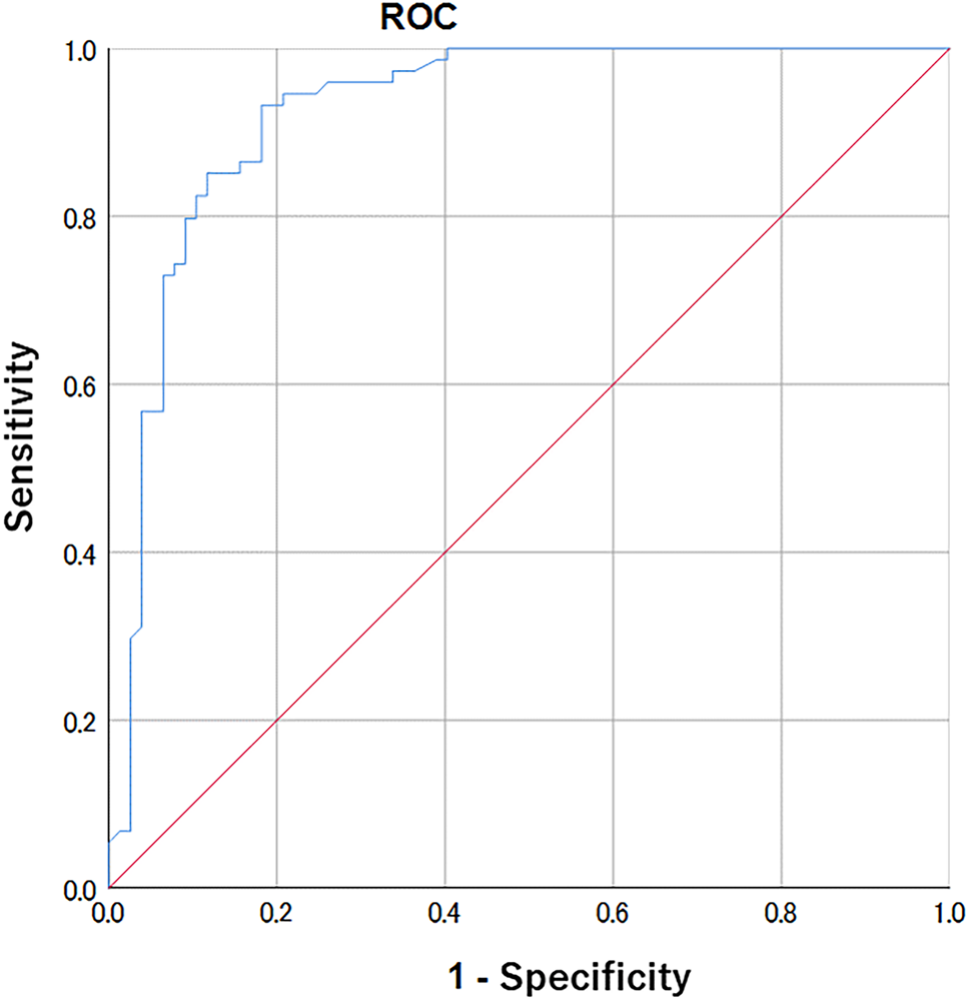
Receiver operating characteristic analysis for mass

**Fig. 3:**
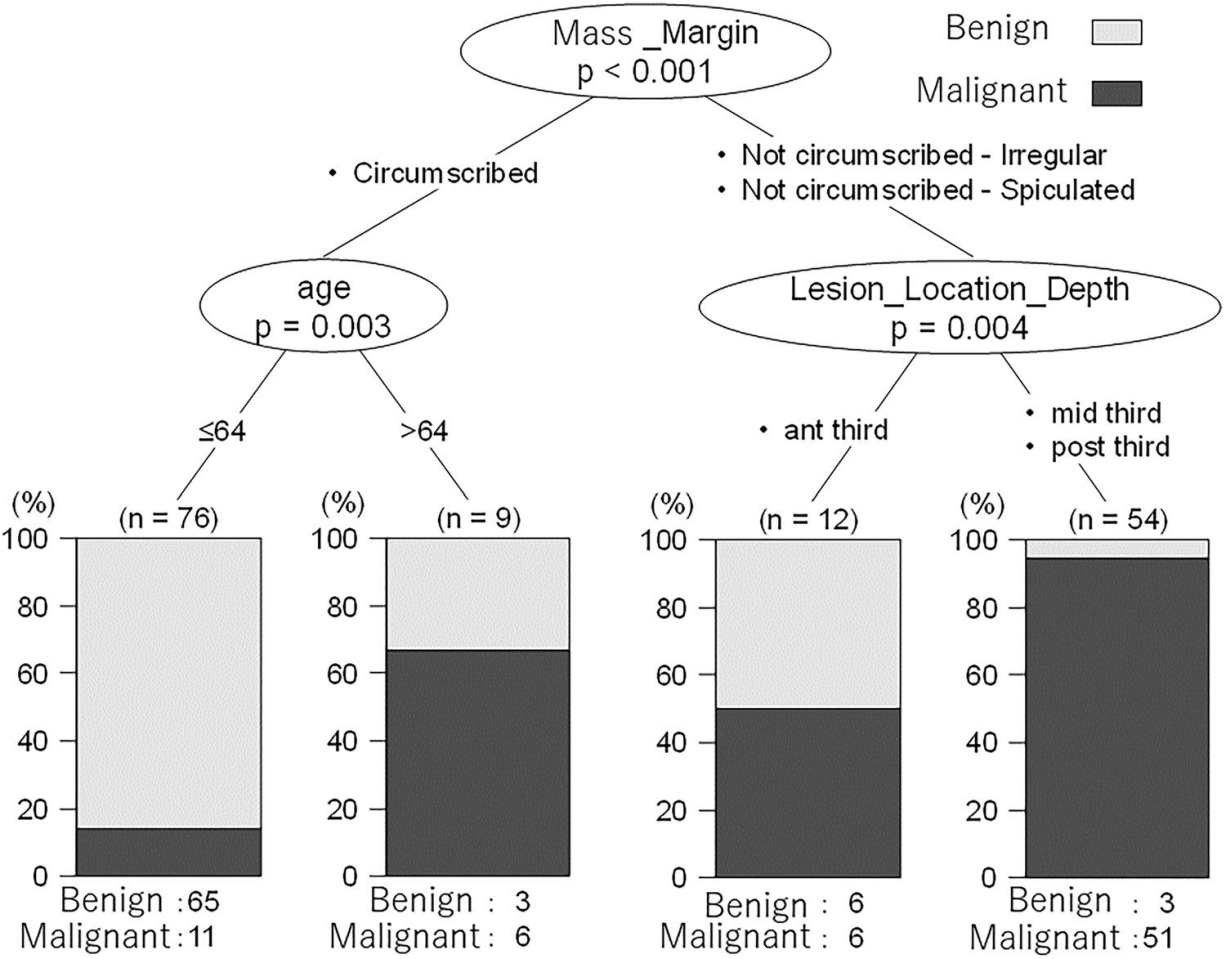
Decision tree analysis for mass

Multivariate logistic regression and decision tree analyses were performed on 151 cases of mass to develop prediction models. Figure 2 and Table 4 show the results of the multivariate logistic regression analysis, and Fig. 3 shows the results of the decision tree analysis.

**Table 4:** Logistic regression analysis for mass

	Partial regression coefficient	Odds ratio	95% confidence interval		*P*-value
			Lower limit	Upper limit	
Age	0.075	1.078	1.023	1.136	0.005
Lesion location—Quadrant (LOQ, UOQ, UIQ, LIQ)	2.703	14.927	1.818	122.573	0.012
Margin (irregular, spiculated)	2.792	16.313	5.109	52.084	0.000
Internal enhancement (heterogeneous, rim enhancement)	1.150	3.157	0.942	10.582	0.062
FGT (fatty, scattered, heterogeneous)	1.132	3.102	0.710	13.541	0.132
Kinetic-delayed phase (plateau, washout)	1.498	4.474	1.527	13.108	0.006

BPE, background parenchymal enhancement; FGT, fibroglandular tissue; LOQ, left outer quadrant; UOQ, upper outer quadrant; UIQ, upper inner quadrant; LIQ, left inner quadrant

^*^Statistical significance was set at *P* < 0.05

According to the multivariate model with logistic regression analysis, old age, lesion location (quadrant: left outer quadrant [LOQ], upper outer quadrant [UOQ], upper inner quadrant [UIQ], or left inner quadrant [LIQ]), margin (i.e., irregular or spiculated), and kinetic-delayed phase (i.e., plateau or washout) significantly increased the risk of malignancy. Moreover, factors, such as internal enhancement (i.e., heterogeneous or rim enhancement) and FGT (i.e., fatty, scattered, or heterogeneous), were included in the model as factors that substantially increased the risk of malignancy, although these factors were not statistically significant. The AUC of the model was 0.925 (95% confidence interval [CI] 0.881–0.970) in the ROC analysis.

According to the decision tree analysis, margin, age, and lesion location (depth) were diagnostic indicators of the model. The sensitivity, specificity, and positive predictive value of this model were 0.770 (95% CI 0.658–0.860), 0.922 (95% CI 0.838–0.971), and 0.848 (95% CI 0.780–0.901), respectively.

**Fig. 4:**
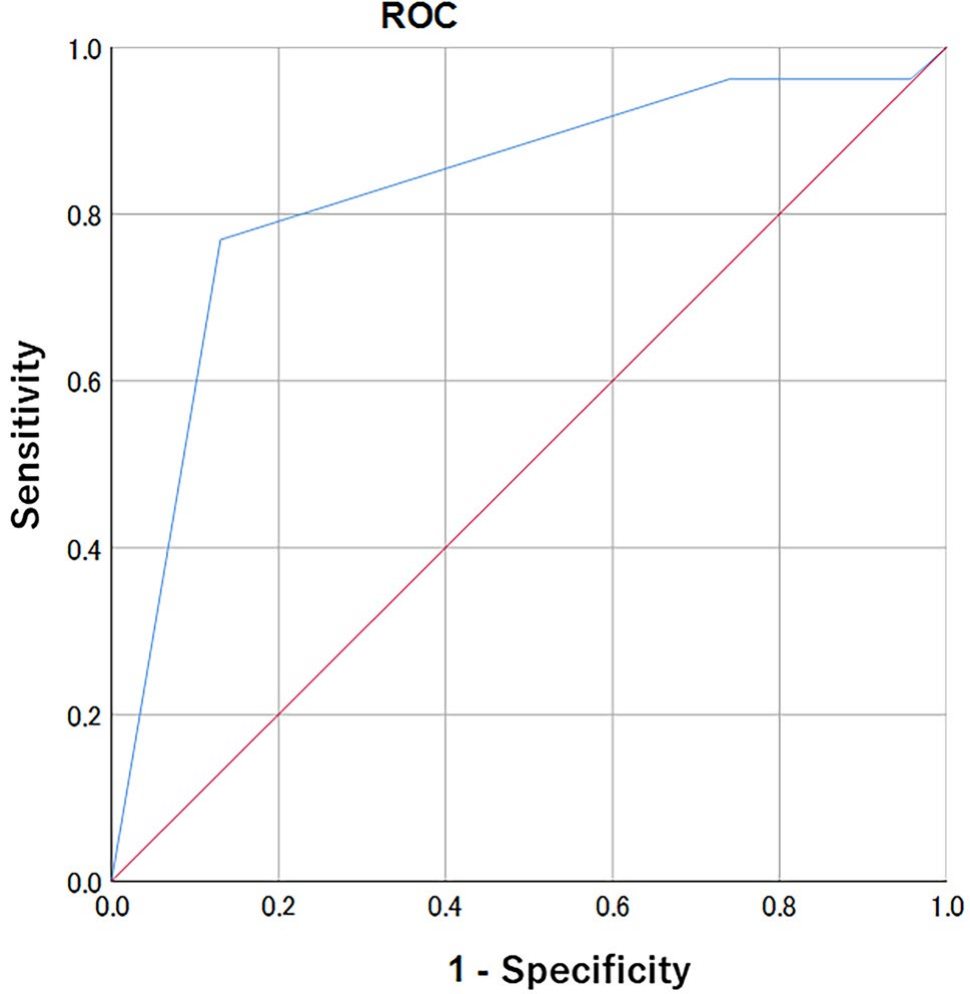
Receiver operating characteristic analysis for non-mass enhancement

**Fig. 5:**
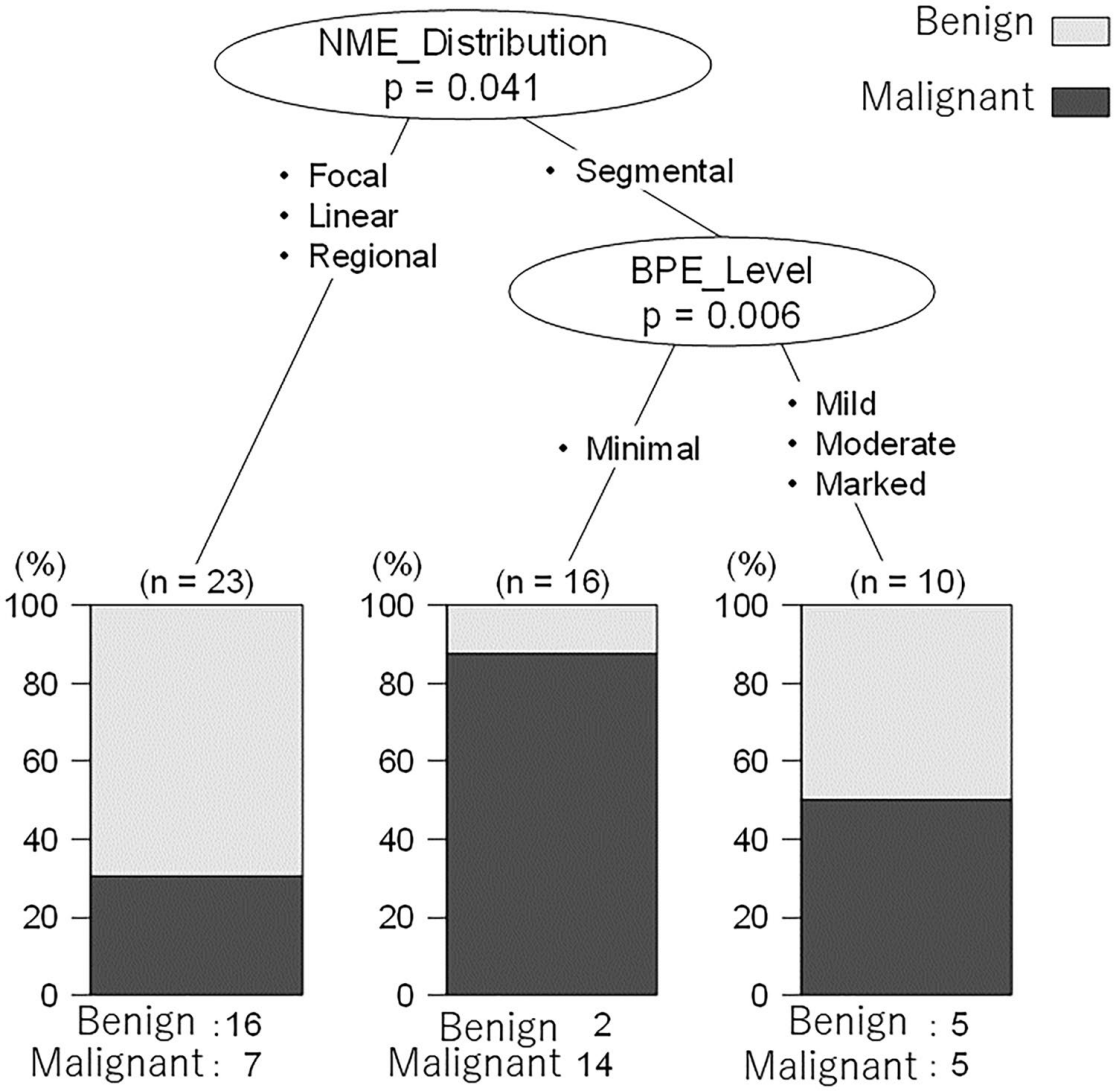
Decision tree analysis for non-mass enhancement

Multivariate logistic regression analysis and decision tree analysis were performed on 49 cases of NME to develop prediction models. Figure 4 and Table 5 show the results of the multivariate logistic regression analysis, Fig. 5 shows the results of the decision tree analysis for NME.

**Table 5:** Logistic regression analysis for NME

	Partial regression coefficient	Odds ratio	95% confidence interval		*P*-value
			Lower limit	Upper limit	
BPE (minimal, mild)	3.014	20.359	1.851	223.963	0.014
Distribution (linear, segmental)	2.355	10.542	2.504	44.378	0.001

^*^Statistical significance was set at *P* < 0.05

According to this multivariate model with logistic regression analysis, the BPE level (i.e., minimal and mild) and distribution (i.e., linear and segmental) significantly increase the risk of malignancy. The AUC of the model was 0.829 (95% CI 0.706–0.951) in the ROC analysis.

According to the decision tree analysis, BPE level and distribution were diagnostic indicators of the model. The sensitivity, specificity, and positive predictive value of this model were 0.538 (95% CI 0.334–0.734), 0.913 (95% CI 0.720–0.989), and 0.714 (95% CI 0.567–0.834), respectively.

## Discussion

Breast MRI detects abnormalities and determines whether these abnormalities are benign or malignant. Achieving a definitive diagnosis using MRI alone is challenging, and additional information is necessary to assess malignancy and ensure proper management. Using terms based on the BI-RADS definition allows for the consideration of findings from the perspective of whether they are more suggestive of malignancy or benignity [16, 17].

In this study, we identified important diagnostic terms to discriminate benign cases from malignant ones by performing univariate and multivariate logistic regression and decision tree analyses, making this a very valuable study.

Univariate analysis revealed significant differences in mass (i.e., shape, margin, internal enhancement, and kinetics) and NME distribution, which are included in the BI-RADS assessment criteria and have been emphasized in previous diagnoses. However, the importance of other factors has not been considered. In particular, the significance of age in the diagnosis was shown to be extremely significant. It is clinically evident that younger individuals often have benign lesions [18]; however, this factor may be less noticeable when examining images alone, making it worthy of attention. Furthermore, in this study, the lesion site (i.e., quadrant and depth) was useful in distinguishing benign cases from malignant ones. Intraductal papilloma, which is a benign tumor, has been shown to have a high incidence in the subareolar and superficial areas [19]. This study included 24 cases of intraductal papilloma, which may have influenced the results. Although lesion location is not included in the BI-RADS diagnostic criteria, it should be a factor that can be used as a reference when making a diagnosis. FGT and BPE levels are factors suggested to be associated with the occurrence of breast cancer, and they have been implicated as factors related to malignancy, indicating their significance in image diagnostics [20, 21]. Although previous studies have reported that lesion size is an independent factor in diagnosing solitary breast masses and incorporating lesion size information into the BI-RADS-MRI 2013 descriptors enables more precise categorization [9], this study did not establish a relationship between lesion size and the likelihood of malignancy. These findings may be influenced by subjective assessments or selection bias, necessitating further investigation.

Multiple regression analysis of masses showed that age, lesion location (quadrant), margin, and kinetic-delayed phase significantly increased the risk of malignancy. The proposed model showed high diagnostic performance in the ROC analysis (AUC of 0.925 [95% CI 0.881–0.970]. In contrast, the decision tree analysis of mass showed that age is useful in the diagnosis process when the margin is circumscribed, and location is useful when the margin is irregular or spiculated. Even if the mass margin is circumscribed, the frequency of malignancy increases with age; therefore, caution should be exercised if the patient is over 64 years of age. If the margin of the mass is irregular or spiculated, the likelihood of malignancy is higher when it is located in the middle or posterior part of the breast. This may be related to previous studies reporting that triple negative breast cancers and breast cancers arising from BRCA2 mutation carriers tend to originate from the posterior portion of the breast [22, 23]. However, this study has not investigated BRCA gene mutations or breast cancer subtypes, so further investigation is needed in the future.

Multiple regression analysis of NME showed that BPE level and distribution significantly increased the risk of malignancy. The model designed for NME had a lower diagnostic performance (AUC of 0.829 [95% CI 0.706–0.951) than the model designed for mass, possibly because of the small sample size. In this study, multiple regression analysis for NME showed that malignancy is suggested when the distribution is segmental and the BPE level is minimal. This may indicate that BPE levels influence the diagnosis of NME. There have been several reports on NME, each reporting the frequency of malignancy according to the BI-RADS lexicon. NME is widely distributed, ranging from 25% to 83.6%, and the frequency of each finding varies [24]. However, there is a limited number of reports focusing on the combination of findings or characteristics specific to benign cases. In this current study, focusing on benign lesions, it has been demonstrated that BPE level and NME are important findings to consider as indicative of benignity in actual readings. In interpreting images, it is crucial not to regard BPE as an abnormal finding. Additionally, in instances of non-mass enhancement, it should be recognized that distributions not characterized as segmental often suggest the possibility of being benign.

Tozaki et al. reported that information on the shape/edge of lesions, heterogeneity within tumors, and kinetic information are useful for distinguishing benign lesions from malignant ones [25]. Moreover, An et al. reported the usefulness of the heterogeneity of internal lesion patterns and low apparent diffusion coefficient values in predicting malignant lesions [26]. However, even in frequently encountered tissues, definitively classifying a benign tumor as benign on MRI can sometimes be challenging. Differentiating intraductal papillomas from ductal carcinomas poses a challenge, and some reports have suggested that a low early-phase enhancement rate and evolution of the DCE-MRI enhancement pattern from homogeneous or heterogeneous enhancement to rim enhancement are more likely to suggest intraductal papilloma [27]. However, even with core biopsy, accurate classification remains difficult. Fibroadenomas, which are known to have high T2 signal intensity, present a challenge in differentiation from phyllodes tumors or mucinous carcinomas, which show similar imaging characteristics [28–30]. In this study, it is suggested that in elderly individuals, especially those aged over 64, even circumscribed masses may have a higher likelihood of malignancy. In younger patients, considering fibroadenomas or phyllodes tumors may enhance diagnostic accuracy, while in older patients, keeping mucinous carcinoma in mind could improve diagnostic precision.

This study was a multicenter collection of only breast MRI images performed using Gadobutrol. The high paramagnetic effect of gadobutrol provides a higher relaxivity, associated with the image quality, as compared to other macrocyclic GBCAs [13, 14]. It has been reported that gadobutolol may reduce the contrast between breast cancer and background parenchyma in premenopausal patients, and that breast cancer patients are characterized as being less likely to "washout" and more likely to "plateau" [15]. In our research, we constructed diagnostic models using multiple regression analysis and decision tree analysis, but it is necessary to be cautious about whether breast MRI images taken with contrast agents other than Gadobutrol can be adapted to this model.

To achieve high diagnostic accuracy, appropriately integrating clinical and imaging features is essential. Combining multiple imaging modalities allows the use of complementary information, enabling a more comprehensive evaluation. Machine learning and deep learning algorithms are gaining attention in medical imaging because they offer the potential to extract patterns from vast amounts of data and construct diagnostic models [31–36]. This can enhance the diagnostic accuracy, providing a more accurate and efficient diagnostic capability. Further research is crucial to determine the true effectiveness of these approaches for breast MRI.

This study has several limitations. First, there is a case selection bias because of the focus on Japanese individuals and the selection of an equal number of malignant and benign cases simultaneously from clinical cases conducted across multiple facilities. Second, nine cases were not pathologically diagnosed as benign lesions among the 100 benign cases. Although this study investigated indicators for distinguishing benign cases from malignant ones, a detailed comparison between individual cases in terms of pathological and imaging findings was impossible. Third, there is a potential for interobserver variability in assessments based on the findings. However, because we used the established BI-RADS terminology, which has already been validated, and adopted a straightforward judgment, the impact of this limitation is considered minimal. Finally, this study lacks a prospective diagnostic evaluation, and further verification is required for different scenarios, such as screening or preoperative settings. Additionally, continuous research to identify new observations is essential.

In conclusion, we conducted a multicenter collaborative study on breast MRI involving Japanese individuals using gadobutrol as the sole contrast agent. Our findings emphasize the significance of incorporating age, lesion location, and BPE level alongside the BI-RADS lexicon morphology features for more accurate determinations, potentially enhancing the differentiation between benign and malignant diagnoses in clinical practice.

## References

[1] Kolb TM, Lichy J, Newhouse JH (2002). Comparison of the performance of screening mammography, physical examination, and breast US and evaluation of factors that influence them: an analysis of 27,825 patient evaluations. Radiology.

[2] Spick C, Szolar DHM, Preidler KW, Tillich M, Reittner P, Baltzer PA (2015). Breast MRI used as a problem-solving tool reliably excludes malignancy. Eur J Radiol.

[3] Yamaguchi K, Schacht D, Sennett CA, Newstead GM, Imaizumi T, Irie H (2013). Decision making for breast lesions initially detected at contrast-enhanced breast MRI. AJR Am J Roentgenol.

[4] Mann RM, Cho N, Moy L (2019). Breast MRI: State of the Art. Radiology.

[5] Tozaki M, Nakamura S (2021). Current status of breast cancer screening in high-risk women in Japan. Breast Cancer.

[6] Peters NH, Borel Rinkes IH, Zuithoff NP, Mali WP, Moons KG, Peeters PH (2008). Meta-analysis of MR imaging in the diagnosis of breast lesions. Radiology.

[7] Mahoney MC, Gatsonis C, Hanna L, DeMartini WB, Lehman C (2012). Positive predictive value of BI-RADS MR imaging. Radiology.

[8] D'Orsi C, Sickles E, Mendelson E, Morris E. Breast imaging reporting and data system. 5th ed. Reston, VA: American College of Radiology; 2013.

[9] Kawai M, Kataoka M, Kanao S, Iima M, Onishi N, Ohashi A, Sakaguchi R, Toi M, Togashi K (2018). The value of lesion size as an adjunct to the BI-RADS-MRI 2013 descriptors in the diagnosis of solitary breast masses. Magn Reson Med Sci.

[10] Istomin A, Masarwah A, Okuma H, Sutela A, Vanninen R, Sudah M (2020). A multiparametric classification system for lesions detected by breast magnetic resonance imaging. Eur J Radiol.

[11] Guirguis MS, Adrada B, Santiago L, Candelaria R, Arribas E. Mimickers of breast malignancy: imaging findings, pathologic concordance and clinical management. Insights Imaging. 2021;12(1):53. 10.1186/s13244-021-00991-x PMC8058137 33877461

[12] Huppertz A, Rohrer M (2004). Gadobutrol, a highly concentrated MR-imaging contrast agent: its physicochemical characteristics and the basis for its use in contrast-enhanced MR angiography and perfusion imaging. Eur Radiol.

[13] Rohrer M, Bauer H, Mintorovitch J, Requardt M, Weinmann HJ (2005). Comparison of magnetic properties of MRI contrast media solutions at different magnetic field strengths. Invest Radiol.

[14] Shen Y, Goerner FL, Snyder C, Morelli JN, Hao D, Hu D, et al. T1 relaxivities of gadolinium-based magnetic resonance contrast agents in human whole blood at 1.5, 3, and 7 T. Invest Radiol. 2015;50:330–8. 10.1097/RLI.0000000000000132 25658049

[15] Tozaki M, Yabuuchi H, Goto M, Sasaki M, Kubota K, Nakahara H (2021). Effects of gadobutrol on background parenchymal enhancement and differential diagnosis between benign and malignant lesions in dynamic magnetic resonance imaging of the breast. Breast Cancer.

[16] Honda M, Kataoka M, Kawaguchi K, Iima M, Miyake KK, Kishimoto AO (2021). Subcategory classifications of Breast Imaging and Data System (BI-RADS) category 4 lesions on MRI. Jpn J Radiol.

[17] Asada T, Yamada T, Kanemaki Y, Fujiwara K, Okamoto S, Nakajima Y (2018). Grading system to categorize breast MRI using BI-RADS 5th edition: a statistical study of non-mass enhancement descriptors in terms of probability of malignancy. Jpn J Radiol.

[18] Youlden DR, Cramb SM, Dunn NA, Muller JM, Pyke CM, Baade PD (2012). The descriptive epidemiology of female breast cancer: an international comparison of screening, incidence, survival and mortality. Cancer Epidemiol.

[19] Eiada R, Chong J, Kulkarni S, Goldberg F, Muradali D (2012). Papillary lesions of the breast: MRI, ultrasound, and mammographic appearances. AJR Am J Roentgenol.

[20] Nara M, Fujioka T, Mori M, Aruga T, Tateishi U (2023). Prediction of breast cancer risk by automated volumetric breast density measurement. Jpn J Radiol.

[21] Watt GP, Thakran S, Sung JS, Jochelson MS, Lobbes MBI, Weinstein SP (2023). Association of Breast Cancer Odds with Background Parenchymal Enhancement Quantified Using a Fully Automated Method at MRI: The IMAGINE Study. Radiology.

[22] Kim WH, Han W, Chang JM, Cho N, Park IA, Moon WK (2015). Location of triple-negative breast cancers: comparison with estrogen receptor-positive breast cancers on MR imaging. PLoS ONE.

[23] Ha SM, Chae EY, Cha JH, Kim HH, Shin HJ, Choi WJ (2017). Association of BRCA Mutation Types, Imaging Features, and Pathologic Findings in Patients With Breast Cancer With BRCA1 and BRCA2 Mutations. AJR Am J Roentgenol.

[24] Kubota K, Mori M, Fujioka T, Watanabe K, Ito Y. Magnetic resonance imaging diagnosis of non-mass enhancement of the breast. J Med Ultrason. 2023;50(3):361–366. 10.1007/s10396-023-01290-2 PMC10353960 36801992

[25] Tozaki M, Igarashi T, Fukuda K (2006). Positive and negative predictive values of BI-RADS-MRI descriptors for focal breast masses. Magn Reson Med Sci.

[26] An YY, Kim SH, Kang BJ. Differentiation of malignant and benign breast lesions: Added value of the qualitative analysis of breast lesions on diffusion-weighted imaging (DWI) using readout-segmented echo-planar imaging at 3.0 T. PLoS One. 2017;12(3):e0174681. 10.1371/journal.pone.0174681 PMC5373600 28358833

[27] Zhu Y, Zhang S, Liu P, Lu H, Xu Y, Yang WT (2012). Solitary intraductal papillomas of the breast: MRI features and differentiation from small invasive ductal carcinomas. AJR Am J Roentgenol.

[28] Yuen S, Uematsu T, Kasami M, Tanaka K, Kimura K, Sanuki J (2007). Breast carcinomas with strong high-signal intensity on T2-weighted MR images: pathological characteristics and differential diagnosis. J Magn Reson Imaging.

[29] Kamitani T, Matsuo Y, Yabuuchi H, Fujita N, Nagao M, Kawanami S (2014). Differentiation between benign phyllodes tumors and fibroadenomas of the breast on MR imaging. Eur J Radiol.

[30] Fujioka T, Kubota K, Kikuchi Y, Tsuchiya J, Tateishi U, Kasaharak M (2018). The feasibility of using 18F-FDG-PET/CT in patients with mucinous breast carcinoma. Nucl Med Commun.

[31] Satoh Y, Imai M, Ikegawa C. Onishi, H. Image quality evaluation of real low-dose breast PET. Jpn. J. Radiol. 2022;40: 1186–1193. 10.1007/s11604-022-01293-y PMC9616787 35612727

[32] Uematsu T, Nakashima K, Harada TL, Nasu H, Igarashi T (2023). Comparisons between artificial intelligence computer-aided detection synthesized mammograms and digital mammograms when used alone and in combination with tomosynthesis images in a virtual screening setting. Jpn J Radiol.

[33] Ueda D, Yamamoto A, Takashima T, Onoda N, Noda S, Kashiwagi S (2021). Visualizing "featureless" regions on mammograms classified as invasive ductal carcinomas by a deep learning algorithm: the promise of AI support in radiology. Jpn J Radiol.

[34] Ishihara M, Shiiba M, Maruno H, Kato M, Ohmoto-Sekine Y, Antoine C (2023). Detection of intracranial aneurysms using deep learning-based CAD system: usefulness of the scores of CNN's final layer for distinguishing between aneurysm and infundibular dilatation. Jpn J Radiol.

[35] Ozaki J, Fujioka T, Yamaga E, Hayashi A, Kujiraoka Y, Imokawa T (2022). Deep learning method with a convolutional neural network for image classification of normal and metastatic axillary lymph nodes on breast ultrasonography. Jpn J Radiol.

[36] Chassagnon G, De Margerie-Mellon C, Vakalopoulou M, Marini R, Hoang-Thi TN, Revel MP (2023). Artificial intelligence in lung cancer: current applications and perspectives. Jpn J Radiol.

